# Is the Pre-Shaping of an Orbital Implant on a Patient-Specific 3D-Printed Model Advantageous Compared to Conventional Free-Hand Shaping? A Systematic Review and Meta-Analysis

**DOI:** 10.3390/jcm12103426

**Published:** 2023-05-12

**Authors:** Ashutosh Kumar Singh, Nikita Khanal, Rajib Chaulagain, Neha Sharma, Florian M. Thieringer

**Affiliations:** 1 Department of Oral and Maxillofacial Surgery, Tribhuvan University Teaching Hospital, Institute of Medicine, Kathmandu 44600, Nepal; dr.ashutosh@iom.edu.np; 2 Department of Population Health Sciences, University of Bristol, Bristol BS8 1QU, UK; drnikitakhanal@gmail.com; 3 Department of Oral Biology, Chitwan Medical College, Bharatpur 44200, Nepal; drrajibchaulagain@gmail.com; 4 Clinic of Oral and Cranio-Maxillofacial Surgery, University Hospital Basel, CH-4031 Basel, Switzerland; florian.thieringer@usb.ch; 5 Medical Additive Manufacturing Research Group (Swiss MAM), Department of Biomedical Engineering, University of Basel, Hegenheimermattweg 167C, CH-4123 Allschwil, Switzerland

**Keywords:** orbital fracture, orbital wall reconstruction, three-dimensional printing, blowout fractures, maxillofacial surgery

## Abstract

This study aimed to perform a systematic review and meta-analysis to compare pre-shaped implants on a patient-specific 3D-printed (3DP) model to manual free-hand shaping (MFS) for orbital wall reconstruction. The PRISMA protocol was followed in this study, and the review was registered in the PROSPERO database (CRD42021261594). A search was conducted in MEDLINE (PubMed), Embase, Cochrane Library, Clinicaltrials.gov, Google Scholar, and the grey literature. Ten articles were included, and six outcomes were analyzed. In total, 281 patients were in the 3DP group and 283 were in the MFS group. The studies had an overall high risk of bias. 3DP models resulted in a better accuracy of fit, anatomical angle reproduction, and defect area coverage. The correction of orbital volume was also superior with statistical significance. There was a higher percentage of the correction of enophthalmos and diplopia in the 3DP group. Intraoperative bleeding and hospital stay were reduced in the 3DP group. The meta-analysis of operative time showed a reduction in the average operative time by 23.58 min (95% CI: −43.98 to −3.19), which was statistically significant (t(6) = −2.8299, *p* = 0.0300). The 3DP models appear advantageous for an accurate orbital wall reconstruction, with fewer complications than those for conventional free-hand-shaped implants.

## 1. Introduction

Post-traumatic orbital wall defect is a frequently managed clinical scenario for craniomaxillofacial surgeons, ophthalmologists, and oculoplastic surgeons [1]. Disconcerting symptoms such as enophthalmos, diplopia, hypoglobus, restricted motility of extraocular muscles, and disturbances in visual acuity often complicate these fractures [2,3]. After the initial management of globe injury and any threat to vision, the goals of management in traumatic orbital wall fractures are to restore the pre-injury orbital volume, thereby correcting the enophthalmos, release any entrapped orbital contents, resolve diplopia (if present), and prevent diplopia in the long-term restoration [4,5].

These goals of repair and restoration are achieved by releasing and reducing trapped orbital contents and reconstructing the orbital wall defect by autogenous grafts or alloplastic biomaterials. As the thin orbital bony walls are not amenable to reduction and fixation, these often require additional support with reconstructive materials [1,6]. These defects have been managed conventionally by various autogenous grafts and alloplastic materials, each with merits and demerits [7,8,9,10]. Autografts have lost to alloplastic materials due to resorption, donor site morbidity, and the need to adapt perfectly to the orbital walls [9,11]. There are a variety of alloplastic implants, but titanium and polyethylene, or a combination of both, are the most widely accepted materials because of their adaptability, tissue inertness, and ease of use [12]. Titanium-based orbital wall implants are the gold standard, but stock titanium mesh or anatomic orbital mesh still require intraoperative cutting, shaping, and adaptation to the individual orbital walls. Although small incisions minimize visible scarring on the face, these can further restrict the direct observation of the orbital fracture. As a result, the contouring and anatomical adaptation of the implant becomes technique-sensitive and relies on the surgeon’s expertise. Multiple trail reinsertions of the implant into the orbit may also be necessary for achieving final adaptation, causing disruption to the sensitive orbital soft tissues and extending the duration of the surgery [13].

Additionally, in the absence of intraoperative imaging or navigational guidance, the surgeon cannot visualize the boundaries of the defect and usually relies on tactile information from instruments or their surgical experience to ensure the final seating of the implant [14,15,16]. As intraoperative imaging and navigational guidance are unavailable in most units, this speculative reconstruction often results in the inadequate adaptation of the implant with a short or malpositioned implant, which often fails to correct the orbital volume [17,18]. This results in revision surgeries, increasing the risk during surgery and further amplifying the patients’ economic burden [19].

Three-dimensional (3D) printing technology (also known as additive manufacturing (AM)) is applied in a variety of craniomaxillofacial scenarios, and its implementation in the reconstruction of orbital wall defects has been energetic [20]. An anatomical model of the fractured orbit or a mirrored image of the contralateral, unaffected orbit can be fabricated using 3D printing for orbital reconstruction. Such 3D-printed, anatomically accurate models, also called biomodels, are used to pre-bend and adapt the orbital implants accurately to the individual orbital anatomy and defect size, which theoretically allows for better clinical and functional outcomes after the reconstruction [21,22]. Although patient-specific implants (PSIs) are available now, these implants are expensive, with longer lead times, and are not widely available globally [23,24]. These 3D-printed models are inexpensive and less time-consuming to produce; hence, they offer value to surgeons and patients [22].

This systematic review intends to compare the results of conventional manual free-hand shaping (MFS) of orbital implants compared to pre-shaped implants on a 3D-printed model (3DP) in reconstructing traumatic orbital wall defects.

## 2. Materials and Methods

### 2.1. Design and Search Strategy

The protocol was registered in the PROSPERO database (International Prospective Register of Systematic Reviews CRD42021261594). The reporting was according to the Preferred Reporting Items for Systematic Reviews and Meta-Analyses (PRISMA Statement) [25].

The PICOS strategy for research hypothesis generation is provided in Table 1. We systematically searched PubMed, Embase, Cochrane Library, Clinicaltrials.gov, Google Scholar, and the grey literature from inception until 15 August 2022. These databases were searched for “orbital fracture,” “reconstruction,” “3D printing,” and variations. The PubMed strategy was adapted for other electronic databases. The detailed search strategy is presented in Appendix A. Reference searching and consultation with experts were performed to include any missed study. Two reviewers (AKS and NK) assessed the eligibility, and disagreements were resolved by a third reviewer (RC).

### 2.2. Eligibility Criteria

The following inclusion criteria were adopted: randomized controlled trials, uncontrolled clinical trials, and prospective or retrospective comparative studies reporting the reconstruction of any human orbital wall defect. Since there is a paucity of clinical trials comparing these two modalities, we included non-randomized comparative studies (NRSI) to obtain a complete update on reported outcomes. Any pre-bent implant on a 3D-printed orbital wall model was considered an intervention, and the conventional free-hand shaping of implants was considered the control. Only published or publication-ahead-of-print full-text articles in the English language were considered. We excluded manufactured PSIs-based orbital reconstructions. The primary outcome of interest was the accuracy of the fit of the implant and the correction of orbital volume. Secondary outcomes were also analyzed, including the correction of pre-operative diplopia, hypoglobus or enophthalmos, operative time (OT), hospital length of stay, cost difference, and complications.

### 2.3. Data Extraction

Two reviewers (AKS, NK) independently performed data extraction, and a third reviewer (RC) acted as a mediator in cases of disagreements. The following information was collected from the studies: first author, country, year of publication, study design, sample size, inclusion and exclusion criteria, intervention, primary and secondary outcomes, follow-up and attrition rate, results, and conclusion. In cases of incomplete information, attempts were made to contact the authors through email to obtain missing outcome data.

### 2.4. Assessment of Risk of Bias

A modified generic version of the Risk of Bias (ROB 2 tool) [26] was used to assess the risk of bias within and across included trials at the study level. The onlinemeta tool based on the R-studio metafor package [27] was used to generate a histogram for the overall risk of bias, and a heatmap was generated for the risk of bias in individual studies based on seven domains (D1 = random sequence generation; D2 = allocation concealment; D3 = blinding of participant and personnel; D4 = blinding of outcome assessment; D5 = incomplete outcome data; D6 = selective reporting; D7 = other sources of bias). Two independent reviewers (AKS, RC) assessed the risk of bias, and any inconsistency was resolved by discussion.

### 2.5. Data Synthesis

A narrative synthesis of outcomes was performed. All statistical analyses were performed using the onlinemeta tool based on the R-Studio metafor package [27]. We evaluated dichotomous outcomes with odds ratios (OR) at 95% confidence intervals (CIs) and continuous outcomes as mean differences at a 95% confidence interval with random effect models. The rank correlation and regression tests using the observed outcomes’ standard error (SE) as the predictor were used to check for funnel plot asymmetry.

## 3. Results

### 3.1. Search Results and Study Characteristics

**Figure 1:**
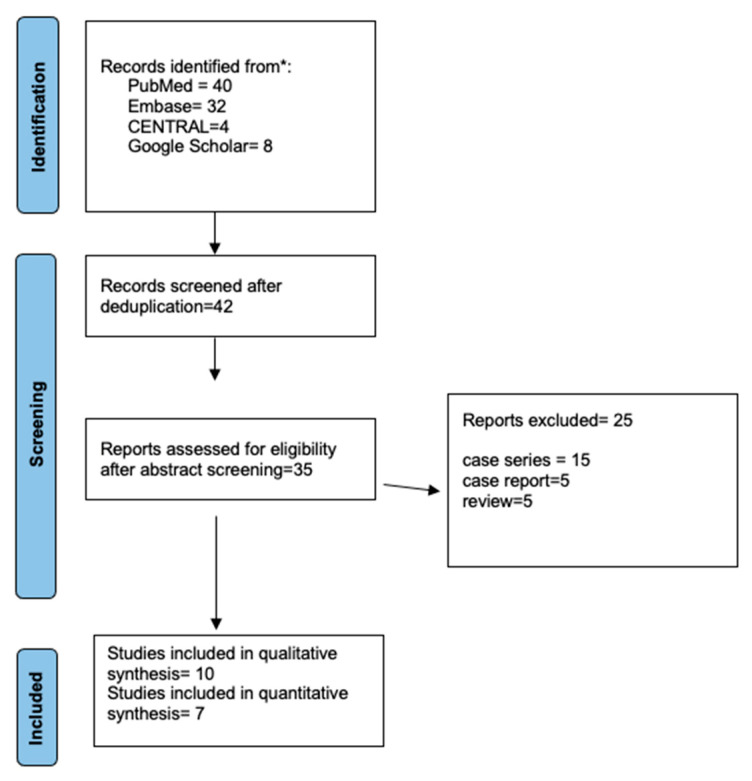
The Prisma flow diagram illustrates the selection process for the articles in the review. * study database-PubMed, Embase, Cochrane Library, Clinicaltrials.gov, Google Scholar, and the grey literature.

After removing duplicates, 42 articles were retrieved; 7 were excluded based on the title and abstract screening. The remaining 35 studies were analyzed in full, and 25 articles were excluded, resulting in 10 studies fulfilling the inclusion criteria. A PRISMA flow diagram for study selection, with an explanation for exclusion at each step, is presented in Figure 1.

The characteristics of the included study are presented in Table 2.

### 3.2. Risk of Bias Assessment

**Figure 2:**
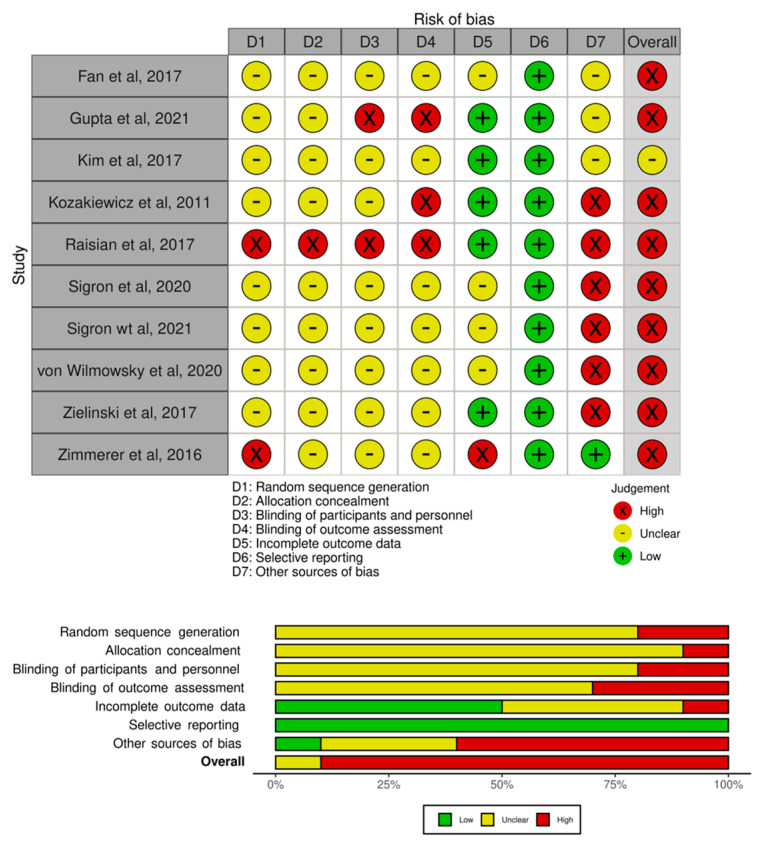
Risk of Bias in individual studies and across the included studies [28,29,30,31,32,33,34,35,36,37].

We performed a systematic risk of bias assessment to identify bias for each included trial. Nine studies [28,29,31,32,33,34,35,36,37] had a high risk of bias, and one study [30] had an unclear risk. The risk of bias in individual studies and across the included studies is summarized in Figure 2.

### 3.3. Outcome and Meta-Analysis

#### 3.3.1. Accuracy of Fit

Kim et al. [30] compared the accuracy of fit by measuring the layout angle and gap length of inserted implants. For medial wall fractures, the average layout angle of the inserted mesh was 3.49° in the 3DP group and 9.03° in the MFS group (*p* < 0.01). For orbital floor fractures, the average layout angle of the inserted mesh was 2.23° in the 3DP group and 4.69° in the MFS group (*p* < 0.01), favoring 3DP-based reconstruction. This study also measured the gap length between the inserted mesh and the fracture defect periphery. For medial wall fractures, the average lengths were 3.1 mm in the 3DP group and 6.37 mm in the MFS group, which was statistically significant (*p* < 0.01). For orbital floor fractures, the average length was 5.21 mm in the 3DP group and 6.64 mm in the MFS group, which was not statistically significant (*p* = 0.12).

Fan et al. [31] measured the accuracy of the implant fit with medical imaging software. Differences in the maximum depth and maximum width between the fracture zone and implant in the control group were significantly higher as compared to the 3DP group (5.60 ± 0.90 vs. 2.51 ± 0.53 maximum width; 4.61 ± 0.89 vs. 2.58 ± 0.46 maximum depth). The difference in the area not covered by the implant was statistically significant and substantially smaller in the 3DP group than in the conventional MFS group. The angle between the medial wall and the floor of the orbit was measured on coronal sections to evaluate the replication of the anatomic angle in comparison with the unaffected eye. The 3DP group presented a more similar anatomic medial-inferior corner angle than the conventional MFS group (*p* < 0.05).

Wilmowsky et al. [34] analyzed the implant fit and accuracy by measuring the congruence of the implant with orbital floor landmarks and graded it from best to worst as complete, good, or acceptable. Accuracy of fit was graded by evaluating the coverage provided by the implant as accurate, too large, and too small. There was no significant difference in congruence between the two groups (*p* = 0.85).

#### 3.3.2. Restoration of Orbital Defect and Volume

Three studies reported this outcome. In the multicenter AOCMF-supported trial by Zimmerer et al. [29], they compared the reconstruction precision between the individualized and stock implants. The individualized implants were either CAD-based or non-CAD-based. CAD-based implants were of two types: patient-specific custom implants (n = 3) and rapid-prototyped resin models (n = 95) of the defect on which pre-shaping and adaptation of the orbital implant were performed for individualization. Non-CAD-based stock implants (n = 100) were manually hand-shaped intraoperatively for reconstruction. Individualized implants had statistically significant precision in the restoration of orbital volume compared to standard preformed plates (variance = 0.6 mL^3^, *p* < 0.001).

In the retrospective analysis by Kim et al. [30], the residual bone defect area in the 3DP group was significantly less in comparison with that of the conventional MFS group (8.03 ± 3.5% versus 18.7 ± 15.41%) for medial wall fractures and (7.14 ± 5.74% versus 12.8 ± 4.92%) for inferior wall fractures, respectively (*p* < 0.01).

Sigron et al. [35] analyzed the pre- and postoperative orbital volume, fracture defect area, and fracture collapse between the 3DP model and MFS group with semi-automated software. There was a statistically significant volume difference in the MFS group (*p* = 0.002) compared to the mirrored unfractured orbit, while in the 3DP group, there was no significant difference in the volume between the pre-operative unfractured orbit and the reconstructed orbit. There was no statistically significant difference in the reduction in the fracture defect area and the postoperative fracture collapse in both groups.

#### 3.3.3. Correction of Orbital Dystopia

Kozakiewicz et al. [28] analyzed the vertical visual disparity (VVD): the globe position at a 30-degree downgaze, primary position, and 30-degree upgaze. They also analyzed the reduction in the extent of diplopia by binocular single vision (BSV) assessment. They reported a significant superiority for the 3DP method over the MFS method in diplopia (*p* = 0.021) and upgazed VVD reduction (*p* = 0.007). The results are not statistically different for the correction of VVD in the downgaze and primary position. There was a statistically significant reduction in the double vision area (*p* = 0.015), improved primary globe position correction (*p* = 0.012), and upgaze (*p* = 0.003) in the long term with the 3DP model. The correction of VVD in downgaze was not different between the two groups.

Zimmerer et al. [29] found no statistically significant difference in diplopia and change in visual acuity over a 12-month follow-up in both groups. Fan et al. [31] reported an absolute difference in residual postoperative enophthalmos and superior sulcus deformity between the reconstructed orbit and the unaffected eye. Both complications were lower in the 3DP group compared to the conventional MFS group. The difference in postoperative enophthalmos was 1.0 ± 0.5 mm vs. 2.5 ± 1.0 mm (*p* < 0.05), and the percentage of superior sulcus deformity in the 3DP group was (6.9%, 2/29) compared to the MFS group (18.5%; 5/27, *p* < 0.05).

Raisian et al. [32] compared the mean enophthalmos at baseline and one week, one month, and four months after surgery. The postoperative enophthalmos was 3.8 ± 0.7, 2.4 ± 0.8, 2.4 ± 0.8, and 2.4 ± 0.8 mm in the conventional MFS group and 2.6 ± 0.8, 0.35 ± 0.4, 0.35 ± 0.4, and 0.35 ± 0.4 mm in the 3DP group. The mean enophthalmos did not differ significantly at baseline between the two groups (*p* = 0.65), while the two groups showed significant differences at one week (*p* = 0.01), one month (*p* = 0.01), and four months (*p* = 0.01).

Sigron et al. [36] evaluated functional ocular symptoms between the two groups with no difference in enophthalmos. There was no statistically significant difference noticed in the improvement of diplopia (*p* = 0.335), restricted ocular motility (*p* = 0.439), or sensory nerve disturbance (*p* = 0.473). Although there was a higher percentage of improvements in all the examined outcomes in the 3DP group, a statistically non-significant result indicates that the better outcomes with 3DP may represent findings due to chance.

Gupta et al. [37] assigned success scores to the reconstruction, enophthalmos correction, diplopia correction, and hypoglobus correction. They also evaluated the quality of life with a questionnaire. There was a statistically significant difference in success scores between the 3DP-based implant and conventional MFS Biopore for reconstruction (9.26 vs. 8.25, *p* = 0.049); enophthalmos correction (10.00 vs. 8.75, *p* = 0.028); and hypoglobus correction (9.00 vs. 7.50, *p* = 0.047). The diplopia correction success score (9.78 vs. 9.06) was insignificant for the groups. However, the scoring was performed by an unblinded scorer and had the potential of a high risk of bias. There was a statistically significant (*p* < 0.01) change in the mean quality of life in the 3DP group compared to the conventional MFS group, as reported by the patients.

#### 3.3.4. Complications

Kim et al. [30] reported a need for revision surgery for enophthalmos and restricted motility in two cases of the conventional MFS group and none in the 3DP group. There was no residual diplopia in the 3DP group after 6 months of follow-up, compared to two cases in the conventional MFS group. Zielinski et al. [33] reported a higher overall intraoperative bleeding in the conventional MFS group compared to the 3DP group (*p* < 0.001). Sigron et al. [36] reported a statistically non-significant reduction in hospital stay (3.8 days) in the 3DP group compared to the conventional MFS group (4.6 days).

#### 3.3.5. Operative Time

**Figure 3:**
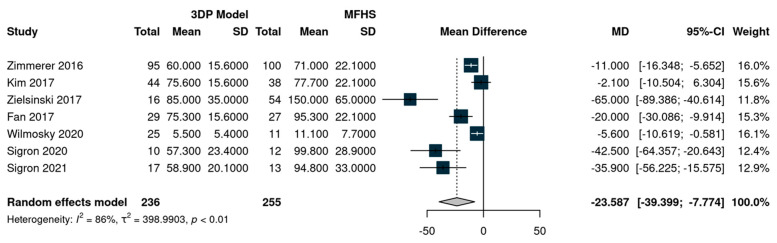
Forest plot for the outcome operative time [29,30,31,33,34,35,36].

The analysis was carried out using the mean difference as the outcome measure. A random-effects model was fitted to the data. Tests and confidence intervals were computed using the Knapp and Hartung method. A total of seven studies were included in the analysis. The estimated average mean difference based on the random-effects model was 23.58 min (95% CI: −43.98 to −3.19), which was statistically significant (t (6) = −2.82, *p* = 0.03). According to the Q-test, the outcome is heterogeneous (Q (6) = 44.35, *p* < 0.0001, tau² = 398.93, I^2^ = 95%). The forest plot is presented in Figure 3.

An examination of the studentized residuals revealed that none of the studies had a value larger than ±2.6901; hence, there was no indication of outliers in the context of this model. According to Cook’s distances, none of the studies could be considered overly influential. Funnel plot analysis and formal statistical tests for publication bias are unreliable, with less than ten included studies; thus, we did not perform a formal analysis for publication bias. All the reported outcomes are presented in Table 3.

## 4. Discussion

The evidence suggests that the use of 3D-printed models to pre-shape and individualize orbital implants results in a superior restitution of orbital volume, defect area coverage, better implant accuracy and fit, a higher percentage of enophthalmos, diplopia and vertical dystopia correction, substantially less operative time, fewer complications, and an increased surgical ease of implant placement in orbit. A recent systematic review [38] reported a similar result, which reported satisfactory outcomes after orbital reconstruction using 3D printing technology. However, the review did not include results from comparative studies between the 3DP model and the MFS technique and also included PSI as 3D printing for orbital reconstruction. In contrast, our review focused on whether the 3DP orbital model allows for better outcomes in post-traumatic orbital reconstruction compared to conventional manual shaping techniques and provides solid evidence.

Some major concerns with free-hand conventional orbital reconstruction were its lack of precision, small implants not covering the complete defect area, or large implants impinging into the orbital apex, requiring a second surgery for prompt removal [39,40]. Though there are anatomic studies that have established the average distance from the orbital rim to the orbital apex, both in the medial wall and floor, to guide the surgeons in cutting and shaping the stock implant, these statistical average distances may not be accurate in some cases due to variance related to age, gender, and race [41,42]. Additionally, it is difficult to reproduce the angle of the medial wall and orbital floor in the coronal section. The improper restoration of the gentle S-shaped curve of the orbital floor results in poorly shaped implants that are unable to reproduce the accurate orbital anatomy. 3DP is a tool that is increasingly being used in medical diagnosis and treatment planning. Because of the orbit’s compact anatomy, surgery within the orbit can be particularly difficult. An alloplastic implant is commonly used to cover the bony defect and prevent tissue herniation into the sinuses during orbital fracture repair. One of the most challenging aspects of such surgery is sizing, contouring, and fitting the implant while preventing collateral damage to orbital tissues [38]. The 3DP model obliviates these inaccuracies, as the implant can be individualized to cover the defect precisely and reproduce the anatomic angles and curves of the orbital walls specific to each patient. This individualized approach creates an esthetically accurate reconstruction with implant adaptation and ensures that the posterior end of the implant sits on the “posterior ledge,” thus correcting the vertical dystopia and enophthalmos [43,44].

One glaring advantage of 3DP models is how individualized implants resulted in the better restitution of the orbital volume and the correction of enophthalmos. The rectification of orbital volume changes is one of the absolute indications for orbital reconstructions, the accuracy of which secures the correction of enophthalmos [45,46]. The reason we found a higher percentage of enophthalmos correction with 3DP models can probably be credited to the complete defect area coverage, which results in the reconstitution of orbital volume to its pre-operative state. On the other hand, diplopia due to the entrapment of orbital contents and restricted extraocular muscle mobility is usually corrected by freeing the entrapped tissues and remains corrected even without complete reconstruction of the defect in small- to medium-sized defects. The contrast is apparent only when the defect is large or multiple-walled, where the risk of prolapse and re-entrapment of orbital tissue increases. The veritable defect area coverage in such cases with 3DP models prevents this relapse and probably is the reason for a lesser proportion of residual diplopia compared to free hand-shaped implants. Two studies in this review have included combined orbital fractures (impure fractures with internal orbital wall and orbital frame fractures) for reconstruction [28,33] in contrast to other studies, which included only isolated orbital fractures. This may introduce heterogeneity in the result; however, we must remember that the treatment protocol of combined orbital fractures dictates that the orbital frame is reduced and fixed first, followed by managing the internal orbital wall defect, which, after the proper and successful reduction in the outer orbital frame, is similar to isolated orbital wall defects clinically. Thus, these results on the orbital wall reconstruction were assimilated in this review.

Even though in the hands of an experienced surgeon, the risk of tissue injury, bleeding, and ocular trauma remains very low, this may not be the same with less experienced trainee surgeons. Frequent manipulations and reinsertions may build on the tissue trauma and bleeding and amplify the risk of preventable ocular trauma [47,48]. This may also affect the postoperative QOL and hospital stay. The results of this review verify that intraoperative bleeding and hospital stay are reduced in the 3DP group, even when the surgery was accomplished by less experienced surgeons, which is exemplary and supports the ethical practice of minimizing patient discomfort with the assistance of available technology. 3DP models can be printed on an inexpensive printer of a reasonable base size. With the advent of automated segmentation software, these models can be made available in every OR around the globe, thus allowing surgeons to use these anatomically accurate models to increase their precision during surgery and reduce patient complications [49]. When 3DP model-based orbital implants were compared with CAD-CAM-based PSIs for orbital defect reconstruction, there was no advantage offered by CAD-CAM PSIs over 3DP model-based implants regarding the ophthalmological outcomes; however, it improved the patient condition post-operatively, mainly due to less intraoperative time and bleeding [33]. Thus, it can be inferred that 3DP models can facilitate similar levels of ophthalmological outcomes compared to PSIs, as PSIs are easier to insert and manipulate because they do not need any intraoperative modifications, as they are fabricated very precisely. Again, the decision to use 3DP models or PSIs is subjective to availability, cost, and urgency.

### Limitations and Strengths

The studies had an unclear risk of bias, most of the studies used various tools to assess the outcomes, and the reporting was subjective. One of the glaring weaknesses in most studies was the unblinded assessment of outcomes, which could introduce selective reporting bias. No study reported cost-effectiveness and technical difficulties in preparing the 3D-printed models.

## 5. Conclusions

Based on the current evidence, we recommend 3DP model-based individualized pre-shaped implants for traumatic orbital wall reconstruction, especially if the defect is multiple-walled or a larger defect where free-hand implants result in less-than-satisfactory outcomes. The decreased operative time is significant, and decreased bleeding with less tissue manipulation during implant placement may result in increased patient comfort and higher QoL after the surgery. However, conclusive evidence-driven change will require comprehensive multicenter trials reporting QOL and patient-reported outcome measures.

## Figures and Tables

**Table 1:** PICOS strategy for the literature search.

Acronym Definition	Description
**(P)** Population	Patients of Any Age with Post-Traumatic Orbital Wall Defect That Required Reconstruction
**(I)** Intervention	Pre-shaped reconstructive implant on an anatomic 3D-printed model of the orbital wall defect
**(C)** Control	Conventional free-hand intraoperative-shaped implant
**(O)** Outcomes	Primary outcome: Fit of the implant, correction of orbital volume and globe position compared to the contralateral uninjured orbit Secondary outcomes: Operative time, complications, the cost difference
**(S)** Study design	Studies in humans, including randomized control trials (RCTs). Uncontrolled clinical trials and prospective or retrospective comparative studies

**Table 2:** Description of study characteristics included in the systematic review.

Author/Year	Study Sample	Country	Study Type	Inclusion Criteria	Follow-Up and Attrition (n)
Kozakiewicz 2011 [28]	MFS = 12 3DP = 12	Poland	Retrospective	Orbital fractures without any coexisting central nervous system or globe injury	12 months, n = 24
Zimmerer 2016 [29]	MFS = 95 3DP = 100	Germany, USA, Spain, Singapore, Austria	Prospective Controlled Multicenter trial	Patients > 18 years with fracture of the orbital floor and/or medial wall not older than 21 days	12 weeks, n = 145
Kim 2017 [30]	MFS = 38 3DP = 44	Korea	Retrospective	Isolated blowout fracture lying unilaterally in the medial or inferior orbit within 1 month from the occurrence	6 months, n = 82
Fan 2017 [31]	MFS = 27 3DP = 29	China	Retrospective	NG	NG
Raisian 2017 [32]	MFS = 5 3DP = 5	Iran	RCT	Orbital bone fracture with at least 2 mm enophthalmos or vertical dystopia or diplopia	4 months, n = 10
Zielinski 2017 [33]	MFS = 54 3DP = 16	Poland	Retrospective	Unilateral side lesion due to trauma, neoplasm, or orbital decompression	NG
Wilmosky 2020 [34]	MFS = 11 3DP = 25	Germany	Prospective	Unilateral fractures of the orbital floor with a large defect requiring mesh	6 months
Sigron 2020 [35]	MFS = 12 3DP = 10	Switzerland	Retrospective	Unilateral isolated orbital wall fracture	NG
Sigron 2021 [36]	MFS = 13 3DP = 17	Switzerland	Retrospective	Unilateral isolated orbital wall fracture requiring surgery with orbital floor mesh	NG
Gupta 2021 [37]	MFS = 16 3DP = 23	India	RCT	Orbital floor fracture with diplopia, enophthalmos, paraesthesia, or a post-traumatic residual deformity	6 months

MFS: Manual free-hand-shaped; 3DP: 3D-printed model-based; ER: Early result; BSV: Binocular single vision; SD: significant. difference; NSD: non-significant difference; RCT: randomized controlled trial; NG: not given.

**Table 3:** Outcomes reported in the included studies.

Study	Accuracy of Fit	Defect Area and Volume	Correction of Orbital Dystopia	Operative Time (min)	Complications
Kozakiewicz 2011 [28]			ER BSV loss (*p* = 0.021) * Reduction in double vision area (*p* = 0.015) Improved primary globe position correction (*p* = 0.012) *		
Zimmerer 2016 [29]		Variance of differences in orbital volume non-CAD based = 0.9 mL^2^ CAD-based = 0.3 mL^2^; (*p* < 0.001)	Sagittal globe position: (*p* = 0.079) Pupillary height: NSD Diplopia: NSD 80% of CAD-based implants inserted by a less experienced surgeon (<10 years of experience) Sensory disturbance: NSD	MFS: 71; 3DP: 60	
Kim 2017 [30]	*Medial wall fracture* Layout angle (°) MFS: 9.03 ± 4.9; 3DP: 3.49 ± 1.97 (*p* < 0.001 *) *Inferior wall fractures* (°) MFS: 4.69 ± 2.51; 3DP: 2.23 ± 1.37; (*p* < 0.001 *) *Gap Length* Medial wall: SD * Inferior wall: NSD	Reduction in the Bone defect area *Medial wall fracture* MFS: 18.7 ± 15.41; 3DP: 8.03 ± 3.5 (*p* < 0.01) *Inferior wall fracture* MFS: 12.8 ± 4.92; 3DP: 7.14 ± 5.74 (*p* < 0.01)		MFS: 77.7; 3DP: 75.6 (*p* = 0.519)	Revision surgery for enophthalmos and restricted motility MFS: 2; 3DP: 0 Residual diplopia > 6 months MFS = 2; 3DP = 0
Fan 2017 [31]	DMW 5.60 ± 0.90 mm; 2.51 ± 0.53 mm (SD) DMD 4.61 ± 0.89 mm; 2.58 ± 0.46 mm (SD) DAR 84.05 ± 20.89 mm^2^; 43.59 ± 9.53 mm^2^ (SD) DAG 12.58 ± 5.04°; 2.82 ± 0.44° (SD)		Postoperative Enophthalmos MFS: 2.5 ± 1.0 mm; 3DP: 1.0 ± 0.5 mm (*p* < 0.05 *)	MFS: 95.37 ± 22.19; 3DP: 75.34 ± 15.68 (*p* < 0.05 *)	
Raisian 2017 [32]			A significant difference in mean postoperative enophthalmos between the two groups after surgery (*p* < 0.01 *) at 1 week, 1 month, and 4 months, respectively		
Zielinski 2017 [33]				Shorter surgery time in patients with individual implants	Higher intraoperative bleeding in patients treated with intraoperative bending titanium mesh (*p* < 0.01) *
Wilmosky 2020 [34]	Accuracy of the implant form of CAD-based pre-bent titanium meshes Accurate = 17 Too large = 6 Too small = 2			MFS: 11.1 ± 7.7; 3DP: 5.5 ± 5.4 (*p* < 0.01 *)	
Sigron 2020 [35]		Mean ± SD absolute volume difference between the conventional and interventional groups MFS: 1.6 ± 1.2 mL; 3DP: 1.0 ± 0.7 mL (*p* = 0.002 *) Fractured area MFS: 408.5 ± 137.5; 3DP: 389.4 ± 135.1 (NSD) Maximum fracture collapse MFS: 6.9 ± 2.3 mm; 3DP: 8.6 ± 5.4 (NSD)		MFS: 99.8 ± 28.9; 3DP: 57.3 ± 23.4 (*p* = 0.001 *)	Postoperative length of hospital stay MFS: 4.6 (3.9) days 3DP: 3.8 (3.0) days (NSD)
Sigron 2021 [36]			With “hybrid” patient-specific titanium meshes, the functional and cosmetic outcome (diplopia, enophthalmos, ocular motility, and sensory disturbance) improved: NSD	MFS: 94.8 ± 33.0; 3DP: 58.9 ± 20.1 (*p* = 0.003 *)	
Gupta 2021 [37]			Success score with material Pre-shaped titanium mesh: 9.26 ± 1.29; Biopore™: 8.25 ± 1.65 (*p* = 0.049) Positive correlation between Success score & QOL score (*p* = 0.034 *) Enophthalmos correction success score for pre-shaped titanium mesh: 10; Biopore™: 8.75 (*p* = 0.028 *) Hypoglobus correction success score for pre-shaped titanium mesh: 9; Biopore™: 7.50 (*p* = 0.047 *) Diplopia correction success score for pre-shaped titanium mesh: 9.78; Biopore™: 9.06; (*p* = 0.200)		

MFS: Manual free-hand-shaped; 3DP: 3D-printed model-based; ER: Early result; BSV: Binocular single vision; CAD: computer-aided design; SD: significant difference; NSD: non-significant difference; CAD: computer-aided design; QOL: quality of life; *: statistical significance.

## Data Availability

The original contributions presented in the study are included in the article; further inquiries can be directed to the corresponding author.

## Appendix A

Search strategy: Keywords and mesh terms used for the search strategy:

Orbital fracture: “orbital fractures”[MeSH Terms] OR (“orbital”[All Fields] AND “fractures”[All Fields]) OR “orbital fractures”[All Fields] OR (“orbital”[All Fields] AND “fracture”[All Fields]) OR “orbital fracture”[All Fields]

Reconstruction: “reconstruct”[All Fields] OR “reconstructability”[All Fields] OR “reconstructable”[All Fields] OR “reconstructed”[All Fields] OR “reconstructible”[All Fields] OR “reconstructing”[All Fields] OR “reconstructional”[All Fields] OR “reconstructive surgical procedures”[MeSH Terms] OR (“reconstructive”[All Fields] AND “surgical”[All Fields] AND “procedures”[All Fields]) OR “reconstructive surgical procedures”[All Fields] OR “reconstruction”[All Fields] OR “reconstructions”[All Fields] OR “reconstructive”[All Fields] OR “reconstructs”[All Fields]

3D Printing: “printing, three-dimensional”[MeSH Terms] OR (“printing”[All Fields] AND “three-dimensional”[All Fields]) OR “three-dimensional printing”[All Fields] OR (“3D”[All Fields] AND “printing”[All Fields]) OR “3D printing”[All Fields].
